# Patient Uncertainty Questionnaire-Rheumatology (PUQ-R): development and validation of a new patient-reported outcome instrument for systemic lupus erythematosus (SLE) and rheumatoid arthritis (RA) in a mixed methods study

**DOI:** 10.1186/s12955-016-0432-8

**Published:** 2016-03-01

**Authors:** Sophie Cleanthous, David Alan Isenberg, Stanton Peter Newman, Stefan John Cano

**Affiliations:** Modus Outcomes, Suite 210b, Spirella Building, Bridge Road, Letchworth Garden City, SG6 4ET UK; Centre for Rheumatology Research, University College London, 5 University Street, London, WC1E 6JH UK; School of Health Sciences, City University, Northampton Square, London, EC1V OHB UK

**Keywords:** SLE, RA, Uncertainty, Psychometrics, Questionnaire-development, Rasch, Cognitive-debriefing, Arthritis, Lupus

## Abstract

**Background:**

An in-depth qualitative exploration of uncertainty in systemic lupus erythematosus (SLE) and rheumatoid arthritis (RA) led to the development of a five-domain conceptual framework of patient uncertainty in these two conditions. The purpose of this study was to develop and evaluate a new patient-reported outcome (PRO) instrument for patient uncertainty in SLE and RA on the basis of this empirically developed conceptual framework.

**Methods:**

Cognitive debriefing interviews were conducted to pre-test the initial items generated on the basis of the preliminary qualitative exploration of patient uncertainty in SLE and RA. Two separate field tests were conducted in five hospital sites to evaluate the measurement properties of the new instrument; the first to identify and form scales, and the second to assess measurement properties of the final version in an independent sample. Psychometric evaluation was conducted in line with the Rasch Measurement Theory (RMT), examining the extent to which sample to scale targeting was satisfactory, measurement scales were constructed effectively and the sample was measured successfully. Traditional psychometric techniques were also used to provide complementary analyses best understood by clinicians.

**Results:**

Pre-testing supported the relevance, acceptability and comprehensibility of the initial items. Findings indicated that the Patient Uncertainty Questionnaire for Rheumatology PUQ-R instrument fulfilled the expectations of RMT to a large extent (including person separation index 0.73 – 0.91). The PUQ-R comprises 49 items across five scales; symptoms and flares (14 items), medication (11 items), trust in doctor (8 items), self-management (6 items) and impact (10 items) which further displayed excellent measurement properties as assessed against the traditional psychometric criteria (including Cronbach’s alpha 0.82 – 0.93).

**Conclusion:**

The PUQ-R has been developed and evaluated specifically for patients with SLE and RA. By quantifying uncertainty, the PUQ-R has the potential to support evidence-based management programmes and research.

**Electronic supplementary material:**

The online version of this article (doi:10.1186/s12955-016-0432-8) contains supplementary material, which is available to authorized users.

## Background

The importance of considering the chronic diseases and their treatment beyond clinical morbidity is increasingly being recognised in many disciplines including rheumatology [1, 2]. In patients with systemic lupus erythematosus (SLE) and rheumatoid arthritis (RA), the patients’ perspective including physical symptoms such as pain and fatigue as well as health-related quality of life (HRQoL) are not always associated with clinical markers of disease [1, 3–5]. Similarly, it is increasingly recognised that patient perceptions and appraisal of one’s condition impact on psychosocial and physical functioning [6] and can further influence patient treatment adherence [7]. One such perception is patient uncertainty which is considered to be particularly relevant in unpredictable conditions like SLE and RA [8–10]. Patient uncertainty has been portrayed as a cognitive stressor with significant implications for patient well-being and management [11, 12].

Cognitive theories view uncertainty as a cognitive state associated with a perceived lack of knowledge and a subjective evaluation or appraisal process which is an inherent part of life [13–15]. It is therefore unsurprising that a disruptive life events like a chronic illness have also been associated with an inevitable sense of uncertainty [8, 16, 17]. The patient uncertainty literature is dominated by the Uncertainty in Illness Theory (UIT) and corresponding instruments [18–20]. These were initially developed in the 1980s to address uncertainty in pre-diagnostic, diagnostic, treatment, and acute illness and was re-conceptualised (RUIT) to address enduring uncertainty in chronic illness [21]. The UIT and RUIT define uncertainty as a cognitive state in which a patient is unable to assign meaning to illness-related events and focus primarily on the sources and appraisal of uncertainty.

Despite providing a very useful generic context for patient uncertainty, qualitative investigations indicate the multidimensional nature of the concept neglected by the UIT and highlight the importance of illness-specific exploration of uncertainty. Specifically qualitative findings in RA, HIV and cancer display how different illness characteristics, for example the illness course, contagiousness, differential treatment advice, and mortality risk, impose different dimensions of uncertainty between different illness groups that can prevail in all aspects of life [22–26].

Our previous in-depth exploration of patient uncertainty in SLE and RA using both patient and rheumatology health-care professionals (HCPs) interviews confirmed this [9]. Patients expressed uncertainty across a variety of domains both directly and indirectly associated with SLE and RA. These were inductively categorised in a five domain framework; including (i) symptoms and prognosis related to uncertainties of symptom and health status interpretation and disease progression; (ii) medical management related to uncertainty of current and future treatment effectiveness as well as uncertainties around doctors’ knowledge and ability to treat a patient; (iii) self-management related to uncertainties around how best to manage and control symptoms and health; (iv) impact related uncertainties related to the potential consequences of disease on all aspects of a person’s life and finally (v) social functioning related to uncertainties around disclosing and handling diagnosis within social circle.

Even though this exploration was conducted in parallel across the two conditions analysis showed that qualitatively the uncertainty domains relevant to SLE and RA patients were overarching hence a common framework was put forward. In line with the heighted clinical complexity of SLE patients reported quantitatively more uncertainties per patient on average; however; younger RA patients reported comparable qualitatively and quantitative uncertainties with SLE patients i.e. uncertainties in the same domains and sub-domains [9].

The manifestation of patient uncertainty in SLE and RA appeared complex, as it comprised different states and not just the inability to assign meaning to illness-related events [19] including a lack of knowledge or understanding, difficulty in interpretation or judgement, unpredictability and the expectation of potential consequences or risks related to the different domains. Patient quotations related to uncertainty were often expressed with an apparent sense of worry and anxiety an issue that was also indicated by HCPs, who further suggested the association of patient uncertainty with treatment adherence and general well-being [9].

This work demonstrated the importance of illness-specific assessment of patient uncertainty as it expanded previous theories [19, 27] by the addition of domains such as impact, comprising issues of family planning and functionality and social functioning, comprising issues of disclosing diagnosis, support and reactions from social circles [9]. Additionally the rheumatology conceptualisation introduced uncertainties related to domains that have been described before such as illness progression and treatment but had not made reference to issues relevant to SLE and RA such as multi-organ involvement unpredictability of flares, medication toxicity and ineffectiveness.

In addition, these findings indicated the insufficiency of existing instruments to adequately capture uncertainty in SLE and RA. Despite their popularity, the UIT instruments were originally developed in the 1980s using data from hospitalised patients targeting acute uncertainty [20]; therefore, content validity in rheumatology is questionable. Furthermore in light of more recent guidelines for patient reported outcome (PRO) development, it is fundamental to support any PRO with empirically derived conceptual framework to ensure that its items are appropriate and comprehensive relative to the concept of interest in the specific context of use to safeguard its content validity [28–30].

In this paper, we take the next steps in the process of developing and evaluating a new PRO instrument for patient uncertainty in SLE and RA. The rising profile of the patient perspective has consequently increased interest in PRO instruments which quantify them [31]. Developing and evaluating PROs which are fit for purpose and provide clinically meaningful and interpretable data is crucial, particularly when numbers generated by them are used to make important decisions about patient care [31, 32]. To address this, more comprehensive and advanced psychometric techniques are increasingly being used and have therefore been chosen in this study.

## Methods

International guidelines and criteria for PRO instruments were used for the development and evaluation process of the PUQ-R [28, 29, 33–35]. The process comprised three stages with independent SLE and RA samples. As the goal was to develop a PRO instrument that could be used across the board of severity in SLE and RA, patients from all disease stages were included in this process. National Research Ethics Committee approval was obtained for this study as well as local Research and Development approval at each of the participating sites.

### Stage 1: Item generation & pre-testing

Item generation involved the development of an exhaustive pool of potential item strings for each domain within the patient uncertainty conceptual framework [9]. Item strings were developed on the basis of patient quotes that were coded as uncertain in the preliminary phase of this study [9]. Following principles of item construction [28, 36, 37], we aimed to have an adequate range of items to cover the breadth of content within each of the five conceptual domains. Items were constructed in lay language using as many of the patients’ own words as possible whilst aiming for brevity and minimal semantic overlap. Item generation was performed in parallel but independently for SLE and RA.

Participants involved in the qualitative interviewing stage of this study [9] were re-invited to participate in the cognitive debriefing interviews. Participants were instructed to complete the initial items whilst thinking aloud to note any queries or problem questions and discuss these with the interviewer [38]. Interviews were digitally recorded and timed. Interview records were reviewed for any issues related with wording ambiguities, relevance and acceptability, in relation to each item, response scale and set of instructions.

### Stage 2: Field test 1

A field test was set up in five hospitals in England: University College Hospital, Kings College Hospital, Royal Blackburn Hospital, Robert Jones and Agnes Hunt Orthopaedic Hospital and Leicester Royal Infirmary. Participants were eligible for participation if they were at least 18 years old, met standard criteria for SLE or RA diagnosis and were fluent in English. Participants with a significant co-morbid diagnosis were excluded. Participants were via two routes; through the post and during outpatient appointments. Personalised letters, standardised instructions and a reminder letter were used to achieve the highest possible response rate [39]. Study materials consisted of a demographics questionnaire and the first draft of the PUQ-R. Examination of these results led to scale modifications and the second draft of the PUQ-R instrument.

### Stage 3: Field test 2

A second field test was set up in four of the participating hospitals (excluding Kings College Hospital). Participant eligibility and recruitment were identical to the first field test. A demographics questionnaire and the second draft of the PUQ-R were administered. This consisted of the five revised scales, including symptoms and flares, medication, trust in doctor, self-management and impact. Rasch analysis was used to evaluate the measurement properties of the PUQ-R scales and to make any necessary additional revisions. Traditional psychometric techniques were then used to assess the measurement properties of the final version of the PUQ-R and complement the psychometric evaluation.

### Stage 2 & 3 statistical analyses

Different psychometric techniques are available for developing and evaluating the scientific rigour of PRO instruments [31]. The modern psychometric paradigm of Rasch Measurement Theory (RMT) [40] offers a mathematical testable model which allows for rigorous testing of measurement properties and therefore leads to the development of instruments which are scientifically sound. A detailed outline of the RMT advantages over traditional psychometrics is presented elsewhere [31, 41].

### Rasch measurement theory analysis

Psychometric evaluation of the PUQ-R scales was performed in line with Rasch Measurement Theory (RMT) using the RUMM2030 software [42]. RMT analysis examines the extent to which observed raw scores match the scores expected by the Rasch model, which indicates the degree to which the summing of scale items results in rigorous measurement (2). The evaluation of a rating scale using Rasch analysis aims to evaluate three broad aspects [32]:

1. How adequate is the sample to scale targeting?
  Scale to sample targeting refers to the comparison between the range of trait (i.e. uncertainty) measured by the scale and the range of the trait measured in the study sample. Targeting was evaluated through examination of the relative distribution of sample and item thresholds as plotted against the same metric scale of logits (the unit of measurement in RMT analysis); where item thresholds reflect the difficulty of each of the multiple response options of each item and the item threshold mean is always set at zero logits [32, 43, 44]. Precision of the person location mean to the item threshold mean indicates adequate targeting [45].
2. To what extent has a measurement scale been constructed successfully?
  Information from four different tests was gathered in order to address this question [41].
  2.1 Do the response categories work as intended?
    Response category thresholds were examined for disordering as the RMT expects them to be ordered in a sequential manner (i.e., “0 = very uncertain”, “1 = somewhat uncertain”, “2 = somewhat certain”,”3 = very certain”) when plotted on the measurement continuum to reflect the decreasing level of uncertainty the responses denote [32, 41].
  2.2 Do the PUQ-R scale items define a single variable?
    RMT expects items within a scale to be cohesive in defining a single measurement continuum [41, 46]. Three “fit” indicators were examined to assess this. Item fit residuals assess whether the item-person interaction is in line with the RMT. Fit residuals reflect the difference between the observed scores and the ones expected by the Rasch model (i.e. observed-expected=residual) and are expected to be distributed between -2.5 to +2.5 [32].
    Chi-square statistics assess whether the item-trait interaction is in line with the RMT. Chi square is a summary statistic computed by dividing the sample into six groups (class intervals) based on their trait (i.e. level of uncertainty). For items to fit the RMT, it is expected that the chi-square probabilities would not be significant (>0.01) [32, 47, 48].
    Item characteristic curves (ICC) are graphical indicators of fit which are used to complement the interpretation of the fit residuals and chi square probabilities [32, 43].
  2.3 Do responses to one item bias responses to others?
    RMT expects that response to an item should not directly influence response to another as this will bias measurement estimates (inflate or deflate reliability). Response dependency is assessed via residual (observed score – expected score= residual) correlations. As the RMT model expects local independence for items, it is also expected that item residuals should be unrelated in order to reflect random error. Residual correlations were used to examine response bias [43, 44] in line with the r>0.30 rule of thumb, but residual correlations below <0.4 were considered as acceptable [49].
  2.4 Is the performance of the scales stable across relevant groups?
    The RMT expects the measurement continuum to perform consistently across different sample groups. Item stability was assessed through differential item functioning (DIF) [32, 41, 50]. DIF explores the relationship between item responses and group membership by examining the observed response differences between class intervals within groups [51]. DIF was assessed between the SLE and RA groups using ANOVA.
3. How has the sample been measured?
  Two indicators were used to examine measurement of the specific sample.
  3.1 Is the sample separated by the PUQ-R scales?
    A scale is expected to detect differences in the levels of trait within a sample and also detect changes in trait levels over time. Within the RMT paradigm the person separation index (PSI) is calculated to assess this [32, 41]. The PSI is computed as the ration of variation of person estimates relative to the estimated error for each person [52]. In other words, the PSI displays how much of the variation in person-location estimates can be associated with random error, where a 0 score indicated all error and a 1 score no error at all [32].
  3.2 To what extent are raw scores linear?
    The extent to which ordinal raw scores approach linear (interval) measurement and their subsequent transformations on an interval scale were assessed. This is important as one point on a scale is not necessarily the same across the breadth of the scale [41, 53]. Considering the stringent mathematical criteria of the RMT minor deviations of raw scores from interval/linear measurement is expected.

### Traditional test theory analysis

To complement the psychometric evaluation the final draft of the PUQ-R scales were further tested to determine whether they fulfilled the widely accepted and used traditional psychometric criteria which are grounded in widely accepted guidelines [28, 33, 35]. Four traditional psychometric properties (Table 1) were assessed using the IBM SPSS Statistics 19 software package. Finally some preliminary construct validity analysis were performed by evaluating differences between the SLE and RA scores across the five PUQ-R scales and convergence of these with other measures of treatment adherence [54], mood [55] and quality of life [56].

**Table 1:** Traditional psychometric properties^a^

Property	Definition	Criteria
Acceptability: Data quality	The extent to which total scores can be computed – data completeness	• Item level missing data <10 %, • scale level missing data <50 %
Acceptability: Targeting	The extent to which the range of uncertainty measured by the scale matches the range of uncertainty in the study sample	• floor & ceiling effects <15 % • skewness statistic range: -1 to 1 • precision of scores and means to scale possible scores & mid-points
Scaling assumptions	The extent to which it is legitimate to sum a set of items, without weighting or standardization to produce a single total score. Summing PUQ-R scores is considered legitimate when (i) items are measured at the same point on the scale (ii) contribute similarly to the variation of the total score; (iii) measure a common underlying construct and (iv) contain similar proportion of information with regard to the construct being measured.	• CITCs ≥0.30 • mean IIC ≥0.30 • ITCs ≥0.30 • item mean scores & standard deviations
Reliability	The extent to which a scale scores are not associated with random error. Scale precision is based on homogeneity of items at a single point in time.	• Cronbach’ s alpha ≥0.7 • homogeneity coefficient • ITCs ≥0.30
Validity	The extent to which a scale measures what it intends to measure. The extent to which a scale measures a single construct was assessed through internal consistency. Item convergent and discriminant validity with an item-total scale correlation criterion of >0.30 for the items’ own scale and a magnitude of > 2 standard errors than other scales.	• Cronbach’s alpha ≥0.70 • ITC between item and own scale: 0.30 - 0.70 • ITC between item and other scale: >2 standard errors of ITC with own scale.

*CITC* corrected item total correlation, *IIC* item-item correlation, *ITC* item total correlation

^a^Psychometric properties are adapted from and explained in more detail in Cano et al 2010 [66]

## Results

### Stage 1: Item development & pre-testing

A total of 82 items were generated for the new instrument called the Patient Uncertainty Questionnaire-Rheumatology (PUQ-R). Items were grouped into five hypothesized scales reflecting the five conceptual domains the items were derived from [9]. Specifically PUQ-R comprised 26 items related to the symptoms and prognosis, 27 items to the medical management, 5 items to the self-management, 18 items to the impact, and 6 items related to the social functioning conceptual framework domain [9].

Even though the volume of uncertainty quotations in the SLE sample was greater, item generation resulted in qualitatively the same breadth of items in both conditions. To this effect, two versions of the PUQ-R were developed, consisting of exactly the same items but a distinctive reference of either lupus or arthritis within the item string. In an attempt to keep the response scale proximal to the latent variable under assessment [28], all items were scored on a 4-point Likert scale reflecting four different degrees of uncertainty.

A total of 20 patients, 10 SLE and 10 RA, were recruited for the cognitive debriefing interviews, the details of which have been described elsewhere [9]. The initial PUQ-R items were well received by participants. No items were omitted, and the completion time ranged from 8 to 30 minutes, including time spent discussing and commenting on items (mean = 18.75, SD = 6.84). A “not applicable” response option was added to address issues of relevance and problem with response scale. The wording of 5 items and two set of instructions was simplified to avoid any ambiguities and one item was split into two to address to separate uncertainty in the workplace and social circle. These changes did not impact on the initial content and structure of the PUQ-R.

### Stage 2: Field test 1

At an average response rate of 60.9 % a total sample of 383 participants was recruited (Table 2). Analyses and interpretation of the RMT psychometric tests resulted in modification and the second draft PUQ-R containing 51 items in total. RMT analysis retained the symptoms and flares, self-management and impact scales whilst splitting the medical management into two scales; medication and trust in doctor. Finally the social functioning items were reduced and merged with the impact scale as they did not perform sufficiently as an independent scale.

**Table 2:** Sample characteristics

	Field test 1			Field test 2		
	Total (*n* = 383)	SLE (*n* = 173)	RA (*n* = 210)	Total (*N* = 279)	SLE (*N* = 165)	RA (*N* = 114)
Age (years)						
Mean (SD)	52.3(16.28)	43.8 (15.2)	59.4 (13.3)	49.93 (14.8)	45.31 (14.3)	56.95 (12.5)
Range	18–86	18–80	23–86	18–84	18–76	20–84
Disease Duration (years)						
Mean (SD)	12.3 (10.8)	11.1 (9.7)	13.3 (11.7)	15.87 (11.2)	16.04 (10.1)	15.60 (12.5)
Range	0.08–54	0.08–39	0.25–54	0.50–52	1–40	0.50–52
Gender n (%)						
Female	320 (83.6)	157 (90.7)	163 (77.6)	245 (87.8)	158 (95.8)	87 (76.3)
Male	63 (16.4)	16 (9.3)	47 (22.4)	34 (12.2)	7 (4.2)	27 (23.7)
Ethnicity n (%)						
White	283 (73.9)	101 (58.4)	182 (86.7)	191 (68.5)	97 (58.8)	94 (82.5)
Black	45 (11.7)	33 (19.1)	12 (5.7)	43 (15.4)	40 (24.2)	3 (2.6)
Indian/Pakistani/Bangladeshi	27 (7.0)	21 (12.1)	6 (2.9)	21 (7.6)	15 (9)	6 (5.3)
Mixed race	11 (2.9)	7 (4.0)	4 (1.9)	6 (2.2)	5 (3.0)	1 (0.9)
Other	11 (2.9)	9 (5.2)	2 (1.0)	11 (3.9)	8 (4.8)	3 (2.6)
Missing	6 (1.6)	2 (1.2)	4 (1.9)	7 (2.5)	–	7 (6.1)

Two items, which displayed significant DIF between the two conditions were retained in the scales but split by DIF and analysed as separately i.e. they were presented in a different order in the SLE and RA version of the symptoms and flares and medication scale to reflect the different level of difficulty each item had for each condition. The performance of the revised improved when re-evaluated within the same sample.

### Stage 3: Field test 2

At an average response rate of 63.4 % a total sample of 279 participants was recruited (Table 2). The second draft of the PUQ-R scales performed consistently well in the first as in the second field test. Further revisions were only made to the symptoms and flares scale which was reduced by two items (Additional file 1). PUQ-R scale psychometric evaluation is presented in line with the methods discussed above, in more length for the RMT analysis and in summary for the traditional psychometrics.

### RMT Analysis: How adequate is the sample to scale targeting?

**Fig. 1:**
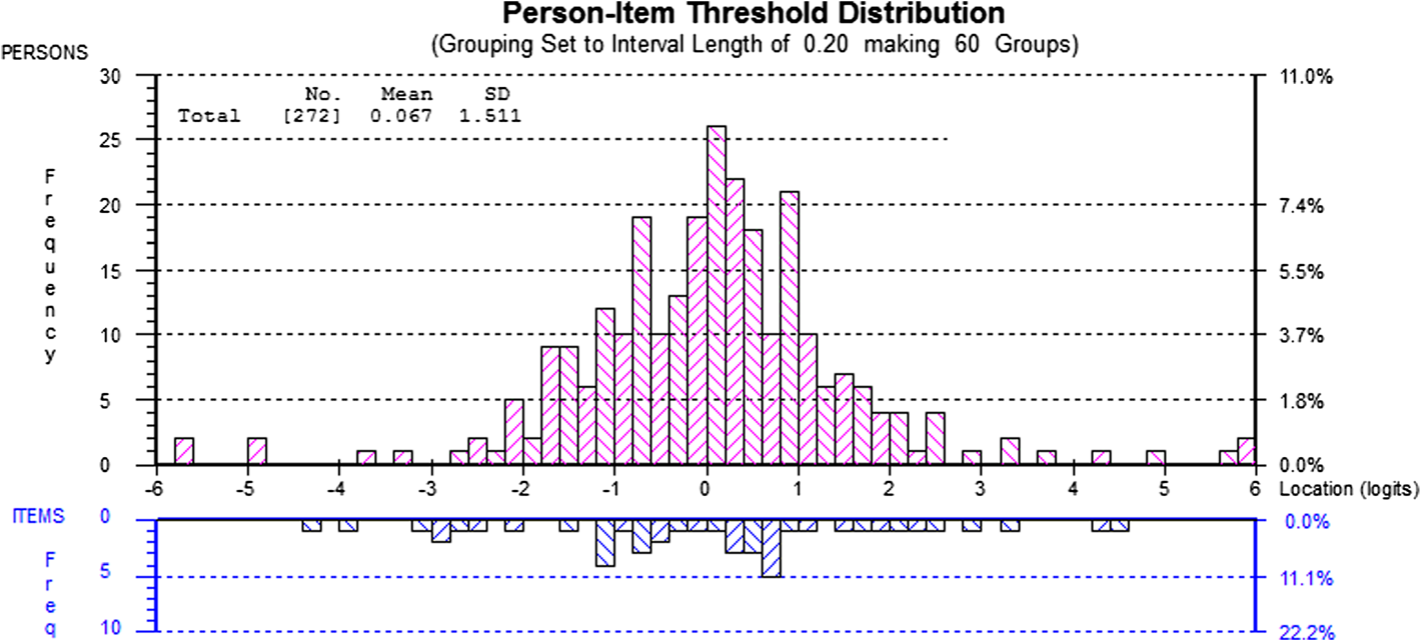
PUQ-R Symptoms & Flares Scale Targeting. The upper histogram (pink blocks) represent the sample distribution for the scale total score whereas the lower histograms (blue blocks) represent the scale item threshold distribution plotted on the same linear measurement continuum. Targeting is satisfactory as the spread of sample and item threshold distributions are well matched. This is also displayed by the person mean location (0.067) which is very close to the item threshold mean location which is always set at zero

**Fig. 2:**
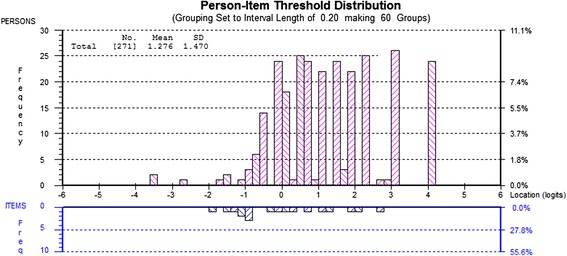
PUQ-R Self-management Scale Targeting. The upper histogram (pink blocks) represent the sample distribution for the scale total score whereas the lower histogram (blue blocks) represent the scale item threshold distribution plotted on the same linear measurement continuum. Targeting is suboptimal. The item thresholds distribution does not match the sample distribution well, as no items are located beyond the +3 logit location. This is also displayed by the person mean location (1.276) which is higher than the item threshold mean location which is always set at zero

PUQ-R scales presented good targeting as the range of uncertainty measured by the scales matched the range of uncertainty in the sample to a satisfactory degree, except for the self-management scale which displayed targeting which was adequate but could stand to be improved. Figure 1 displays the sample-to-scale distributions for the symptoms and flares scale displaying very good targeting. In comparison, the self-management scale targeting graph (Fig. 2) indicates many person measurements located on the right hand side of the continuum, signifying respondents with the highest scores i.e. less uncertainty, who are not covered by the scale items. This can also be deducted by the self-management person mean score (1.276) which is the highest of all PUQ-R and the one furthest away from the item mean score (which is also set at zero logits). Person location mean scores for the remaining scales were 0.067, 0.675, 0.845 and -0.246 for the symptoms and flares, medication, trust in doctor and impact scales respectively.

### RMT analysis: to what extent has a measurement scales been constructed successfully?

**Fig. 3:**
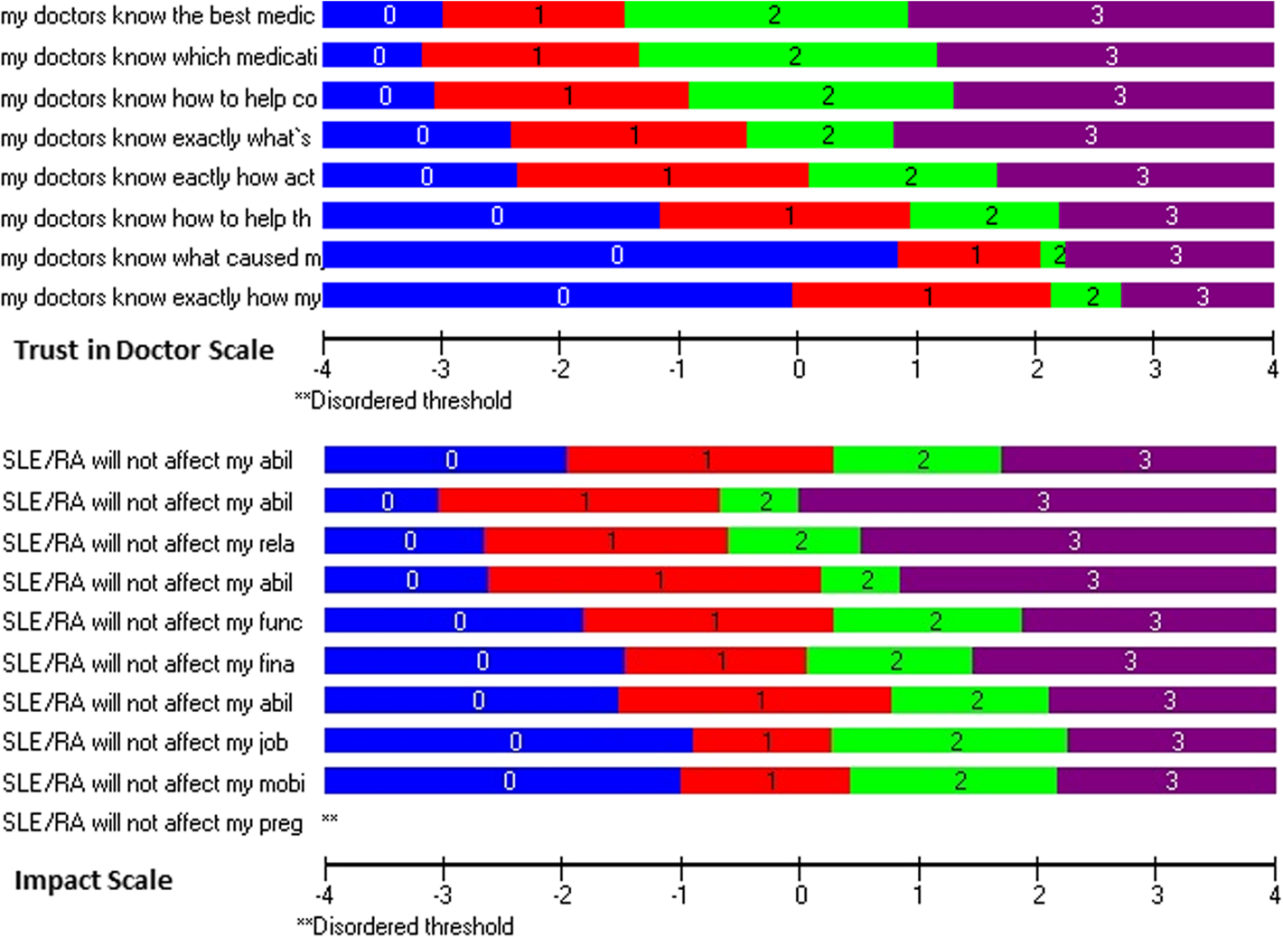
PUQ-R Scale Threshold map Examples. Threshold maps for all PUQ-R scales. The x-axis represents the measurement continuum of the trait (uncertainty), with decreasing levels from left to right. The y-axis shows each of the items response categories “Very Uncertain” labeled as 0; “Somewhat Uncertain” labeled as 1; “Somewhat Certain” labeled as 2 and “Very Certain” labeled as 3. Thresholds for items are missing and replaced with ** if they are disordered, i.e. response categories do not appear in a consecutive increasing order in relation to the construct (x-axis)

The PUQ-R scales were constructed successfully as findings displayed minor deviations from the RMT expectations. All item response categories were ordered in sequence apart from three out of forty-nine items; item 34 of the self-management scale that was consistently disordered in the first field test and items 15RA and 49 of the medication and impact scales evaluated for the first time in this field test. The response category “somewhat uncertain” was problematic for items 34 and 15RA and the “somewhat certain” for item 49. Examples of threshold maps are illustrated in Fig. 3.

Item goodness of fit was excellent for three of the PUQ-R scales as only one item of the trust in doctor and three items of the impact scale displayed statistical misfit with fit residual outside the recommended criterion and significant chi square probabilities (Table 3). However, when misfit was assessed graphically via the ICCs (graphs not presented can be obtained from authors), misfit was marginal for items 41 and 45 of the impact scale. More evident misfit was displayed by item 33 of the trust in doctor and item 49 of the impact scale which both underestimated the trait presented scores higher than expected at lower end of the continuum (i.e. less uncertainty for the less able persons) and lower scores than expected at the higher end of the continuum (i.e. more uncertainty for more able persons).

**Table 3:** PUQ-R measurement scales item-level data

Item String		Loc.	SE	Fit Res.	Chi Sq.	Prob.	res. r	DIF ANOVA (df = 1)		
								MS	F	Prob.
PUQ-R Symptoms & Flares Scale (PSI = 0.91)										
1	straight away	−1.82	0.11	−2.00	14.09	0.00	0.33	5.93	8.68	0.00
2	specific symptoms	−1.64	0.10	−0.98	3.39	0.34	<0.30	1.31	1.57	0.21
3	everyday symptoms	−1.22	0.10	−0.40	5.48	0.14	0.33	2.51	2.90	0.09
4	serious symptoms	−1.12	0.09	0.40	0.87	0.83	<0.30	0.90	0.93	0.34
5	getting older	−1.04	0.09	0.80	2.75	0.43	<0.30	1.91	1.91	0.17
6	side-effects	−0.87	0.09	2.35	4.48	0.21	<0.30	0.36	0.31	0.58
7	all different	−0.80	0.09	−0.89	10.36	0.02	<0.30	0.08	0.10	0.76
8SLE	symptom triggers	−0.39	0.10	2.09	11.03	0.01	0.37	0.00	0.00	1.00
9	flare type	0.36	0.08	−0.24	2.99	0.39	<0.30	2.09	2.33	0.13
10	symptom timing	0.46	0.09	0.84	1.79	0.62	0.37	2.27	2.24	0.14
8RA	symptom triggers	0.80	0.14	2.05	7.49	0.06	<0.30	0.00	0.00	1.00
11	future effect	1.21	0.09	1.44	3.75	0.29	0.42	0.29	0.26	0.61
12	flare timing	1.39	0.10	0.11	7.08	0.07	0.32	8.93	10.13	0.00
13	flare severity	2.11	0.11	−0.38	1.67	0.64	0.51	2.40	2.77	0.10
14	flare frequency	2.57	0.12	−1.12	5.15	0.16	0.51	0.67	0.88	0.35
PUQ-R Medication Scale (PSI = 0.91)										
15RA	need medication	−1.54	0.17	−0.96	1.81	0.61	0.32	0.00	0.00	1.00
16	help symptoms	−0.91	0.10	−0.75	1.38	0.71	0.44	27.30	39.46	0.00
17	controls condition	−0.56	0.09	−1.23	5.21	0.16	0.49	1.02	1.34	0.25
15SLE	need medication	−0.52	0.11	2.56	31.68	0.00	0.49	0.00	0.00	1.00
18	stronger dose	−0.24	0.09	−0.68	3.32	0.34	0.48	2.01	2.44	0.12
19	will help symptoms	−0.20	0.10	0.63	1.80	0.61	0.51	0.22	0.23	0.63
20	need additional	−0.08	0.09	−1.61	5.27	0.15	0.52	0.25	0.33	0.57
21	need alternative	0.05	0.09	0.16	0.21	0.98	0.52	0.66	0.71	0.40
22	will control	0.07	0.10	0.43	2.44	0.49	0.51	0.04	0.04	0.84
23	will need stronger	1.22	0.09	−0.30	2.18	0.54	0.75	4.38	5.13	0.02
24	will need additional	1.32	0.10	0.66	4.21	0.24	0.75	2.98	3.24	0.07
25	will not alternative	1.37	0.09	1.05	2.26	0.52	0.62	0.29	0.28	0.60
Item		Loc.	SE	Fit Res.	Chi Sq.	Prob.	res. r	DIF ANOVA (df = 1)		
								MS	F	Prob.
PUQ-R Trust in Doctor Scale (PSI = 0.73)										
26	best dose	−1.16	0.11	−2.75	16.24	0.00	0.71	0.76	1.29	0.26
27	which medication	−1.10	0.11	−2.66	22.06	0.00	0.71	1.43	2.49	0.12
28	help physical	−0.88	0.10	−1.15	10.30	0.02	<0.30	3.69	5.09	0.02
29	what’s wrong	−0.67	0.10	0.37	4.05	0.26	<0.30	1.95	2.16	0.14
30	physically active	−0.19	0.10	−1.59	8.02	0.05	<0.30	0.13	0.18	0.67
31	help non-physical	0.67	0.09	1.28	1.41	0.70	<0.30	0.21	0.21	0.65
32	future progress	1.61	0.09	1.63	1.52	0.68	<0.30	4.56	4.45	0.04
33	cause	1.72	0.08	4.23^a^	36.72	0.00^b^	<0.30	0.24	0.17	0.68
PUQ-R Self-management Scale (PSI = 0.86)										
34	questions	−0.52	0.10	0.93	10.46	0.02	<0.30	0.55	0.59	0.44
35	symptom report	−0.49	0.11	−0.56	3.69	0.30	<0.30	4.20	5.60	0.02
36	test results	0.11	0.09	0.91	3.65	0.30	<0.30	6.49	7.34	0.01
37	activities to avoid	0.15	0.09	0.10	1.84	0.61	<0.30	0.06	0.07	0.79
38	how to manage	0.32	0.10	−1.11	9.91	0.02	<0.30	3.19	4.61	0.03
39	help control	0.43	0.09	0.93	7.34	0.06	<0.30	2.50	2.81	0.10
PUQ-R Impact Scale (PSI = 0.87)										
40	education	−1.24	0.16	0.56	1.20	0.75	<0.30	0.48	0.47	0.49
41	relationship	−0.91	0.10	2.11	15.09	0.00^b^	0.32	9.20	8.89	0.00
42	children	−0.53	0.13	1.66	5.36	0.15	0.32	4.41	4.21	0.04
43	plan life	0.01	0.10	−1.92	13.60	0.00	<0.30	1.57	2.39	0.12
44	finances	0.02	0.09	1.10	1.73	0.63	<0.30	0.00	0.00	0.97
45	functionality	0.12	0.10	−3.60^a^	14.73	0.00^b^	<0.30	8.29	16.21	0.00^c^
46	exercise	0.46	0.10	0.48	1.46	0.69	<0.30	0.55	0.61	0.44
47	mobility	0.54	0.09	−1.36	7.06	0.07	<0.30	5.21	7.45	0.01
48	job prospects	0.54	0.11	−0.58	5.71	0.13	<0.30	0.71	0.94	0.33
49	pregnancy	0.99	0.16	3.31^a^	27.10	0.00^b^	<0.30	0.63	0.32	0.58

*Loc* item Location; *SE* standard error; *res. r* residual correlation; *DIF* differential item functioning

^a^fit residuals outside the recommended range is −2.5 to +2.5

^b^Chi-square probability significant after Bonferroni adjustment at *p* < 0.01

^c^DIF by patient group (SLE Vs RA) significant after Bonferroni adjustment at <0.01

Some response bias was revealed in the final version of the medication scale items evaluated for the first time in the second field test (Table 3). Another two item pairs displayed significant response bias; the symptoms and flares items 13 and 14 and the trust in doctor items 26 and 27 and produced high residual correlation coefficients. The performance of the scale items was stable across SLE and RA as only one item (item 45) displayed significant statistical DIF between the two conditions. Assessing this graphically revealed that observed scores for the SLE sample for item 45 related to functionality, were higher than expected, and lower than expected for the RA sample.

### RMT analysis: How has the sample been measured?

All PUQ-R scales produced high PSI (073 – 0.91), thus confirming their ability to separate the sample (Table 3). The linearity of measurement was evaluated graphically by plotting the raw scores on a graph against interval measurement (graphs not presented can be obtained from authors). Graphs for all PUQ-R scales displayed an expected sub-optimal S-shaped relationship raw scores and interval measurement and scores were used to calculate a transformed 0-100 interval scoring for each of the five scales.

### Traditional psychometrics

The PUQ-R scales satisfied the traditional psychometric analysis criteria (Table 1). PUQ-R scale acceptability (quality & targeting) was excellent with very low percentages of scale-level missing data and no floor and ceiling effects or any statistical skewness (Table 4). Scaling assumptions were further met as the range of corrected item total correlations (CITCs) and mean item-to-item correlation (IIC) for all scales laid above the 0.30 criterion. PUQ-R scales mean scores were also very close to the actual mid-point. Findings also greatly supported the PUQ-R scales reliability with Cronbach’ s alpha coefficient well above the 0.70 criterion for all scales, which further satisfied the item-level validity criteria. Preliminary examination of the PUQ-R scales construct validity showed significant relationships between different PUQ-R scales and measures of treatment compliance, depression, anxiety, physical and mental quality of life (Table 5). Means comparison between the SLE and RA sample revealed a significant difference only in the symptoms and flares scales with higher scores for the SLE patients (t = -4.40, df = 277, p = 0.00) and non-significant differences across all other scales. This was in line with heightened clinical complexity of SLE and previous qualitative findings [9].

**Table 4:** Traditional psychometrics scale-level results

	Data quality	Scaling assumptions				Targeting			Reliability		Item convergent – discriminant validity: ITC range				
	Missing data %	Possible range (mid- point)	Actual score range	Mean (SD)	CITC range	Floor effect %	Ceiling effect %	Skewn.	Cronbach’s alpha	IIC mean	1	2	3	4	5
Symptoms & flares	7.52	14–56 (35)	14–56	35.29 (7.99)	0.44–0.69	0.72	0.72	−0.35	0.90	0.40	0.54–0.73	0.05–0.20	0.12–0.34	0.16–0.45	0.02–0.25
Medication	4.30	11–40 (27.50)	11–44	30.96 (6.77)	−0.35–0.71	0.72	3.94	−0.15	0.90	0.60	0.02–0.18	0.46–0.77	0.23–0.40	0.25–0.33	0.04–0.43
Trust in doctor	2.15	8–32 (20)	8–32	22.37 (4.90)	0.40–0.71	0.72	1.43	−0.24	0.86	0.61	0.32–0.14	0.23–0.49	0.56–0.78	0.39–0.20	0.24–0.49
Self-management	3.58	6–24 (15)	6–24	18.80 (3.68)	0.53–0.67	0.72	8.60	−0.61	0.82	0.60	0.18–0.48	0.19–0.44	0.26–0.40	0.67–0.78	0.04–0.21
Impact	2.51	10–40 (25)	10–40	24.95 (8.15)	0.39–0.79	1.43	0.36	−0.13	0.93	0.73	0.00–0.24	0.11–0.42	0.20–0.43	0.04–0.29	0.49–0.84

*CITC* corrected item total correlation, *Skewn* skewness statistic, *IIC* item-item correlation, *ITC* item total correlation

**Table 5:** Preliminary construct validity analysis (Pearson correlations)

PUQ-R	CQR	HADS-A	HADS-D	PCS	MCS
SLE					
Symptoms & flares	–	–	–	–	–
Medication	0.22*	−0.18*	−0.29**	0.28**	0.22**
Trust in doctor	0.33**	−0.23**	−0.36**	0.21**	0.20**
Self-management	–	−0.28**	−0.21**	–	0.16*
Impact	0.18*	−0.38**	−0.52**	0.47**	0.31**
RA					
Symptoms & flares	0.26*	–	–	–	–
Medication	0.31**	–	–	0.19**	0.19*
Trust in doctor	0.39**	–	–	–	–
Self-management	–	−0.20*	−0.24*	0.20**	0.20*
Impact	–	−0.43**	−0.57**	0.35**	0.34**

*CQR* Compliance Questionnaire Rheumatology

HADs-A Anxiety; HADs- D; Depression PCS physical component scale (SF-36); MCS mental component scale (SF-36)

*p < 0.05

**p < 0.01

## Discussion

The PUQ-R is a PRO instrument developed using comprehensive qualitative methodology, incorporating the input of patients with SLE and RA and rheumatology HCPs, rigorous psychometric techniques in line with best practice guidelines [28, 29, 33, 35] and rheumatology outcome-recommendations [57, 58]. It quantifies patient uncertainty in SLE and RA across five different domains; symptoms and flares, medication, trust in doctor, self-management and impact (Additional file 1). These were suggested as important aspects of the SLE and RA illness experience by patients themselves in a preliminary study [9] which uncovered aspects of patient uncertainty not covered by older generic theories and instruments [18–20, 27].

The empirical content development of the PUQ-R [9] supports its relevance for patients with SLE and RA and the subsequent pre-testing of items ensures that the instrument is acceptable and appropriate for patients. The extensive quantitative RMT psychometric analysis supported the suitability of use of the PUQ-R scales [41] which also displayed excellent measurement properties when assessed against the traditional psychometric criteria [33]. Preliminary construct validity examinations indicated negative association of different uncertainty aspects with other important patient outcomes.

Although these findings support the PUQ-R’ s measurement properties, developing an instrument using rigorous methodology is an on-going process [28, 29]. An RMT psychometric evaluation provides a vehicle for evidence-based scale improvement by signifying areas of sub-optimal performance. In this respect, the RMT psychometric evaluation of the PUQ-R satisfies all criteria for its initial use, but further highlights areas needing improvement including the sample-to-scale targeting for the self-management scale and item dependency for the medication scale that would benefit from further empirical testing.

Finally, the raw ordinal total scores of the PUQ-R scales did not reflect interval measurement. However, this was an expected finding as raw scores are ordinal and unsurprisingly have unequal intervals. The advantage of RMT analysis is the ability to obtain implied interval measurements [59] which can be used to calculate a transformed 0-100 interval scoring for sub-sequent use. This issue is not always addressed in PRO instruments; however, it is highly important, particularly when interpreting scores from a total ordinal scale which have unequal intervals [41]. This analysis therefore benefits from the provision of interval-level transformed scoring.

Patient uncertainty has been linked with unfavourable outcomes in SLE and RA [9, 26, 60, 61] and in chronic illness in general [11, 12]. The PUQ-R is the first instrument developed to quantify patient uncertainty specific to SLE and RA and also the first instrument to the authors’ knowledge to quantify uncertainty as a multi-dimensional concept across different domains. The PUQ-R could therefore be used in studies exploring the impact of patient perceptions on outcomes of disease such as HRQoL, physical symptoms like pain and fatigue as well as treatment adherence [1, 4–7, 9].

Several self-management interventions in chronic illness and rheumatology have drawn from the bio-psychosocial model and other social cognition theories to improve moderating variables of chronic illness, such as patient perceptions, self-efficacy and coping [1, 62]. Preliminary construct validity analysis indicates that higher uncertainty across different but not all domains are associated with lower treatment adherence, higher levers of depression, anxiety and poorer HRQoL.

If these relationships are established patient uncertainty could be targeted as a moderating variable in self-management interventions to evaluate whether it is amenable and whether it can subsequently influence other patient outcome. For example, whether decreasing levels of uncertainty in relation to the trust patients have in their doctors would improve treatment adherence in the SLE sample, or whether decreasing levels of medication and impact uncertainty would improve depression levels in RA and HRQoL in both conditions. Such could be potential uses of the PUQ-R instrument in patient research and management.

Lastly it is important to acknowledge potential limitations of this work and areas for future work. The sample size for both field tests was sufficient considering the general “rule of thumb” recommending 5 to 10 participants per scale item [63]; however, there was room for improvement as far as the response rate is concerned. Response rates exceeded the reported 60 % average response rate in medical and nursing surveys [64, 65]; nevertheless, a post-hoc investigation revealed that changes in study design could have improved this.

Screening for all three stages of this study did not limit the sample to a specific disease stage as the intention was develop a PRO instrument applicable across all ranges of disease. Future work should aim to evaluate whether levels of disease severity influence the levels of patient uncertainty expressed by patients, as well as to establish psychometric performance of the PUQ-R across all stage of SLE and RA disease using a clinical measure of disease. Finally, a more extensive exploration of construct validity, minimally clinically important difference and responsiveness of the PUQ-R should follow suing longitudinal data and clinical measures of disease which were not available during this study.

## Conclusions

The PUQ-R was developed and evaluated in line with best practice guidelines [28, 29, 33–35] rheumatology outcome-recommendations [57, 58] using comprehensive methodology and a large amount of patient input. Therefore, a new instrument like the PUQ-R enhances the field of health measurement in rheumatology, by offering the opportunity to quantify in a valid and meaningful way, aspects of the patient perspective within SLE and RA. This study contributes a scientifically rigorous instrument to SLE and RA health measurement and further offers a useful template for the rigorous step-wise development and validation of PRO instruments.

## Additional file

Additional file 1: Patient Uncertainty Questionnaire - Rheumatology (PUQ-R). (PDF 425 kb)

## Abbreviations

CITC: corrected item total correlation
DIF: differential item functioning
HCP: health care professional
HRQoL: health related quality of life
ICC: item characteristic curve
IIC: item-to-item correlation
PSI: person separation index
PUQ-R: patient uncertainty questionnaire-rheumatology
RA: rheumatoid arthritis
RMT: rasch measurement theory
SLE: systemic lupus erythematosus
