# Supplementary material for: Patient Uncertainty Questionnaire-Rheumatology (PUQ-R): development and validation of a new patient-reported outcome instrument for systemic lupus erythematosus (SLE) and rheumatoid arthritis (RA) in a mixed methods study
Source: Health Qual Life Outcomes. 2016 Mar 1;14:33. doi: 10.1186/s12955-016-0432-8 (PMC4772528; doi:10.1186/s12955-016-0432-8)
Supplement: Additional file 1: — Patient Uncertainty Questionnaire - Rheumatology (PUQ-R). (PDF 425 kb) [file 12955_2016_432_MOESM1_ESM.pdf]

Copyright @ 2014 University College London.

Patient Uncertainty Questionnaire - Rheumatology (PUQ-R)

This questionnaire includes questions about issues related to your arthritis/lupus

(*Depending on population of administration the word arthritis or lupus is used through the questionnaire*).

Please answer each question according to **HOW CERTAIN OR UNCERTAIN** you are about each of these issues.

Please answer all of the questions. If you are not sure, make your best guess.

For additional information please contact sophie.cleanthous@gmail.com

Copyright @ 2014 University College London.

The following questions relate to your arthritis/lupus symptoms. Thinking about your arthritis/lupus, how certain are you that you understand which symptoms are arthritis/lupus symptoms and which are not? Please **circle** the option that best describes **how certain** you are about the following statements.

	**Very Uncertain**	**Somewhat Uncertain**	**Somewhat Certain**	**Very Certain**
I can tell straight away when I am experiencing an arthritis/lupus symptom.	1	2	3	4
I can tell apart everyday arthritis/lupus symptoms from flares.	1	2	3	4
I can tell which symptoms are specific to arthritis/lupus.	1	2	3	4
I can judge how serious my arthritis/lupus symptoms are.	1	2	3	4
I can tell apart arthritis/lupus symptoms from the natural symptoms of getting older.	1	2	3	4
I can tell arthritis/lupus symptoms apart from side-effects caused by the medication I am taking.	1	2	3	4
I know all the different symptoms related to my arthritis/lupus.	1	2	3	4
I know when to expect an arthritis/lupus symptom.	1	2	3	4
I know how long my arthritis/lupus symptoms last.	1	2	3	4
I know what triggers my arthritis/lupus symptoms.	1	2	3	4

For additional information please contact sophie.cleanthous@gmail.com

Copyright @ 2014 University College London.

Think about the state of your arthritis/lupus and how well you are at the moment. Please **circle** the option that best describes **how certain** you are about the following statement.

	**Very Uncertain**	**Somewhat Uncertain**	**Somewhat Certain**	**Very Certain**
My arthritis/lupus is under control at the moment.	1	2	3	4

Think about the medications you are taking for your arthritis/lupus at the moment. Please **circle** the option that best describes **how certain** you are about the following statements.

	**Very Uncertain**	**Somewhat Uncertain**	**Somewhat Certain**	**Very Certain**	**Not Applicable**
I need the medication I am currently taking for my arthritis/lupus.	1	2	3	4	na
The medications I am taking are helping my arthritis/lupus symptoms.	1	2	3	4	na
The medication I am taking is controlling my arthritis/lupus.	1	2	3	4	na
I do NOT need a stronger dose of medication for my arthritis/lupus.	1	2	3	4	na
I do NOT need additional medication for my arthritis/lupus.	1	2	3	4	na
I do NOT need alternative medication for my arthritis/lupus.	1	2	3	4	na

For additional information please contact sophie.cleanthous@gmail.com

Copyright @ 2014 University College London.

How well do you think your rheumatology doctor(s) know your arthritis/lupus? Please **circle** the option that best describes **how certain** you are about the following statements.

	**Very Uncertain**	**Somewhat Uncertain**	**Somewhat Certain**	**Very Certain**	**Not applicable**
My doctor(s) know which medication(s) and dose(s) are the best for me.	1	2	3	4	na
My doctor(s) know which medication will work best for me.	1	2	3	4	na
My doctor(s) know how to help me control the physical aspects of my arthritis/lupus.	1	2	3	4	na
My doctor(s) know exactly what's wrong with me.	1	2	3	4	na
My doctor(s) know exactly how physically active I should be.	1	2	3	4	na
My doctor(s) know how to help me with the non-physical aspects of my arthritis/lupus (e.g. feeling low).	1	2	3	4	na
My doctor(s) know what caused my arthritis/lupus.	1	2	3	4	na
My doctor(s) know exactly how my arthritis/lupus will progress in the future.	1	2	3	4	na

For additional information please contact sophie.cleanthous@gmail.com

Copyright @ 2014 University College London.

Think about the management of your arthritis/lupus and how well you understand it. Please circle the option that best describes how certain you are about the following statements.

	**Very Uncertain**	**Somewhat Uncertain**	**Somewhat Certain**	**Very Certain**	**Not applicable**
I understand my doctor's/nurse's questions, comments and recommendations.	1	2	3	4	na
I understand what my medical test results mean.	1	2	3	4	na
I know which symptoms I need to report to my doctor.	1	2	3	4	na
I know which types of physical activity I should avoid.	1	2	3	4	na
I know exactly how to manage my arthritis/lupus.	1	2	3	4	na
There are things I can do to help control my arthritis/lupus (e.g. avoid or recover from flares).	1	2	3	4	na

Thinking about how people react to your arthritis/lupus, how certain are you about revealing your diagnosis? Please **circle** the option that best describes **how certain** you are about the following statement.

	**Very Uncertain**	**Somewhat Uncertain**	**Somewhat Certain**	**Very Certain**	**Not Applicable**
I can confidently reveal my arthritis/lupus diagnosis to others (e.g. social circle & workplace)	1	2	3	4	na

Copyright @ 2014 University College London.

Think about the future development of your arthritis/lupus and how easy it is for you to predict it. Please **circle** the option that best describes **how certain** you are about the following statements.

	**Very Uncertain**	**Somewhat Uncertain**	**Somewhat Certain**	**Very Certain**
I know that my arthritis/lupus will flare-up at some time in the future.	1	2	3	4
I know what type of flare-ups I will experience.	1	2	3	4
I can predict when I will experience a flare-up.	1	2	3	4
I can predict how arthritis/lupus will affect me in the future.	1	2	3	4
I can predict how severe my flare-ups will be.	1	2	3	4
I can predict how often I will experience a flare-up.	1	2	3	4

Think about how your arthritis/lupus will develop in the future. Please **circle** the option that best describes **how certain** you are about the following statements.

	**Very Uncertain**	**Somewhat Uncertain**	**Somewhat Certain**	**Very Certain**
The symptoms of my arthritis/lupus will stay the same in the future.	1	2	3	4

	**Very Uncertain**	**Somewhat Uncertain**	**Somewhat Certain**	**Very Certain**
The severity of my arthritis/lupus will stay the same in the future.	1	2	3	4

For additional information please contact sophie.cleanthous@gmail.com

Copyright @ 2014 University College London.

	Very Uncertain	Somewhat Uncertain	Somewhat Certain	Very Certain
I can predict how well I will be in the future.	1	2	3	4

Think about the effectiveness of your medication in the future. Please **circle** the option that best describes **how certain** you are about the following statement.

In the future…	Very Uncertain	Somewhat Uncertain	Somewhat Certain	Very Certain	Not Applicable
The medication I am taking will help my arthritis/lupus symptoms.	1	2	3	4	na
The medication I am taking will control my arthritis/lupus.	1	2	3	4	na
I will NOT need a stronger dose of medication for my arthritis/lupus.	1	2	3	4	na
I will NOT need additional medication for my arthritis/lupus.	1	2	3	4	na
I will NOT need alternative medication for my arthritis/lupus.	1	2	3	4	na

Think about what additional treatments for your arthritis/lupus you might need to have in the future. Please **circle** the option that best describes **how certain** you are about the following statement.

	Very Uncertain	Somewhat Uncertain	Somewhat Certain	Very Certain
I will NOT need to have surgery related to my arthritis/lupus in the future.	1	2	3	4

For additional information please contact sophie.cleanthous@gmail.com

Copyright @ 2014 University College London.

Think about what effect (if any) the medication you are taking for arthritis/lupus could have on your body. Please **circle** the option that best describes **how certain** you are about the following statement.

	**Very Uncertain**	**Somewhat Uncertain**	**Somewhat Certain**	**Very Certain**	**Not Applicable**
The medication I am taking will NOT cause any severe side effects in the future.	1	2	3	4	na

Think about the arthritis/lupus-care team that looks after you (e.g. rheumatology doctors or nurse). Please **circle** the option that best describes **how certain** you would feel about the following statement.

	**Very Uncertain**	**Somewhat Uncertain**	**Somewhat Certain**	**Very Certain**
It would not be a problem if my arthritis/lupus care team (e.g. different doctor or hospital) changed.	1	2	3	4

Think about how your arthritis/lupus might develop. How certain are you that you will be able to manage your arthritis/lupus in the future? Please **circle** the option that best describes **how certain** you are about the following statement.

	**Very Uncertain**	**Somewhat Uncertain**	**Somewhat Certain**	**Very Certain**
I will be able to manage my arthritis/lupus in the future.	1	2	3	4

For additional information please contact sophie.cleanthous@gmail.com

Copyright @ 2014 University College London.

Think of how arthritis/lupus may affect aspects of your personal life. Please **circle** the option that best describes **how certain** you are about the following statements.

	**Very Uncertain**	**Somewhat Uncertain**	**Somewhat Certain**	**Very Certain**	**Not applicable**
Arthritis/lupus will NOT affect my ability to plan my life (e.g. housework)	1	2	3	4	na
Arthritis/lupus will NOT affect my ability to complete my education	1	2	3	4	na
Arthritis/lupus will NOT affect my relationship with my partner or a future partner.	1	2	3	4	na
Arthritis/lupus will NOT affect my ability to care for my children.	1	2	3	4	na
Arthritis/lupus will NOT affect my functionality (e.g. ability to dress, do the house chores).	1	2	3	4	na
Arthritis/lupus will NOT affect my finances.	1	2	3	4	na
Arthritis/lupus will NOT affect my ability to exercise.	1	2	3	4	na
Arthritis/lupus will NOT affect my job prospects.	1	2	3	4	na
Arthritis/lupus will NOT affect my mobility (e.g. my ability to walk).	1	2	3	4	Na
Arthritis/lupus will NOT affect my pregnancy.	1	2	3	4	Na

For additional information please contact sophie.cleanthous@gmail.com

Copyright @ 2014 University College London.

Thinking about practical and emotional support, how certain are you that your family and loved ones will support you in the future? Please **circle** the option that best describes **how certain** you are about the following statements.

	**Very Uncertain**	**Somewhat Uncertain**	**Somewhat Certain**	**Very Certain**
My family and loved ones will help me manage my arthritis/lupus.	1	2	3	4

For additional information please contact sophie.cleanthous@gmail.com
